# Service delivery and coverage in primary healthcare in a community-health project in Ibadan, Nigeria

**DOI:** 10.4102/phcfm.v6i1.545

**Published:** 2014-02-19

**Authors:** Kabiru K. Salami, William R. Brieger

**Affiliations:** 1 Department of Sociology, University of Ibadan, Nigeria; 2 Department of International Health, Bloomberg School of Public Health, Johns Hopkins University, USA

## Abstract

**Background:**

Standard health-service delivery aimed toward improving maternal and child health status remains elusive in Nigeria because of inaccuracies in data documentation leading to a lack of relatively stable evidence.

**Objectives:**

Through a community-health project, this study tested the accuracy of record keeping in primary healthcare services in nine clinics run in Ibadan, Nigeria.

**Methods:**

A validation exercise was performed through a sample of the 10 most recent names extracted from three registers maintained by each clinic.

**Results:**

A review of the register covering a period of four years showed a steady increase in: fully-immunised children, registration for antenatal care during the first trimester of pregnancy, the number of women who attended antenatal care at least three times, the overall number of women who booked for antenatal care and women who delivered in Eniosa Community-Health Project facilities over the four-year period. It was possible to trace 86% of those selected from the antenatal care register, 88.9% of those from the birth register and 81.1% of those from the immunisation register. Four women who should have been included for antenatal care, seven who had delivered (but were not in the register) and 13 who reportedly received immunisation but were not listed were found during the validation exercise.

**Conclusion:**

This study concludes that the names appearing in the register are likely to represent valid events, but that the registers did not capture all such events in the community.

## Abstrait

**Validation de la fourniture de service et de la couverture en soins de santé primaires: l'expérience tirée d'un projet de santé communautaire à Ibadan, au Nigeria**

**Contexte:**

La fourniture de services de santé normaux visant à améliorer les conditions de santé de la mère et de l'enfant reste rare au Nigeria en raison d'imprécisions dans les données documentées, qui entraînent un manque de preuves suffisamment solides.

**Objectifs:**

Au moyen d'un projet de santé communautaire, cette étude a testé la précision des traces écrites laissées par les services de soins de santé primaires dans neuf cliniques d'Idaban, au Nigeria.

**Méthodes:**

Un exercice de validation a été effectué sur un échantillon des 10 noms les plus récents tirés de trois registres tenus par chaque clinique.

**Résultats:**

Un examen du registre couvrant une période de quatre ans a montré une augmentation régulière du nombre d'enfants totalement vaccinés, de l'inscription aux soins prénatals pendant le premier trimestre de la grossesse, le nombre de femmes venues au moins trois fois pour des soins prénataux, le nombre total de femmes qui se sont inscrites aux soins prénatals et les femmes ayant accouché dans les locaux du Projet de santé communautaire Eniosa sur la période de quatre ans. Il était possible de retrouver 86% de celles choisies dans le registre de soins prénatals, 88.9% de ceux du registre de naissance et 81.1% de ceux du registre des vaccinations. Quatre femmes qui auraient dû être incluses aux soins prénataux, sept qui avaient accouché (mais n’étaient pas dans le registre) et 13 qu'on disait avoir été vaccinées mais n’étaient pas sur la liste furent trouvées lors de l'exercice de validation.

**Conclusion:**

Cette étude conclut qu'il est probable que les noms apparaissant dans le registre reflètent des événements valides, mais que les registres n'avaient pas enregistré tous ces événements dans la communauté.

## Introduction

### Background

Despite the declaration and commitment asserted by the Nigerian national health policy (NHP) that ‘Primary health care (PHC) shall remain the basic philosophy and strategy for national health development’^1^ and that ‘PHC is the key to attaining the goal for all people of Nigeria’, studies on evaluation of health indices have shown slow progress toward agreed health goals^2^ and steady deteriorating conditions have been shown during every health system evaluation carried out in Nigeria.^3^ Lack of a standard healthcare system is one major factor in sub-Saharan Africa,^2^ with Nigeria ranking 187th for its health systems performance out of 191 member States.^4, 5^ Access to healthcare is restricted by geographic-, socioeconomic- and/or financial- and sociocultural factors.^6, 7^ In many countries in sub-Saharan Africa a substantial proportion of all health services are provided by the private sector.^8–11^ Even in government facilities where there are qualified personnel^12^ and relatively cheaper services, Batega^13^ reported shorter opening hours, irregular drug supply or unavailable drugs and the poor attitude of health workers. Other studies have reported on the factors that determine the health behaviour of patients in various contexts, including physical-, socioeconomic-, cultural- and political factors.^14–16^

Access to a PHC facility is projected as being a basic social right,^17^ but proximity to the nearest facility,^18^ socioeconomic status of the individual,^14, 19^ cultural beliefs and practices^20^ and a low level of trust and confidence in the health provider have been a major barrier, particularly in rural areas, thereby hindering the access to services. Both non-use and late use have also been reported in studies, especially amongst mothers of under-five children in Nigeria,^21^ as being responsible for the high rate of maternal mortality in Africa. Several studies have reported various factors, including place of residence, breastfeeding status, place of delivery, access to postnatal care and maternal age and education, as influencing child health and survival.^22–24^ The consequences of maternal-health challenges in Africa have undermined social- and economic development and are more devastating in resource-poor settings.^25^ The overall implication is that the highest infant- and maternal mortality in the world has been reported in sub-Saharan Africa, with the region accounting for half of the 8.8 million under-five year mortality globally in 2008.^17^ In addition, an estimated 1000 deaths per 100 000 live births occurred in Africa, with one in 20 women dying as a result of pregnancy-related conditions in Africa compared with one in 4000 deaths amongst European women.^26^ A recent report by a consortium of United Nations agencies attested to a sharp drop in maternal mortality in East Asia, but a slow one in Africa in the last two decades, with maternal deaths falling to about 287 000 in 2010.^27^ The very quick drop in the maternal mortality in East Asia, as opposed to the slow drop in Africa, was attributed to an increase in contraception and antiretroviral drugs for mothers with HIV and to a greater numbers of births attended by specialists.^27^ Although there is ample evidence of success stories worldwide, there is a need to validate the hospital-based reporting in Nigeria in order to arrive at reliable and valid data. The Eniosa Community-Health Project (ECHP) in Lagelu local government area (LGA) validated the major services and coverage in the project registers from the end users at the end of four years of the existence of the project. The ECHP was the first non-profit, private charitable organisation to run PHC services in Nigeria and aims to achieve a sustainable healthcare system in rural Nigerian communities. This study was part of the performance assessment of a community-health project in Nigeria.

## Eniosa Community-Health Project

The Eniosa Community-Health Project is one of several health and development activities in Nigeria. This community-health project was supported in Nigeria by the Tulsi Chanrai Foundation, a charitable, philanthropic and non-profit private voluntary organisation. The Chanrai family has been in business in Nigeria since 1891 – well over a century. The Foundation provided PHC services in the rural areas of Oyo, Osun and Kaduna States at the time of this assessment. The first such PHC system was called the ECHP, established in September 1997 and situated in the Lagelu and Akinyele Local Government Areas of Oyo State. It was designed to address the health needs of the community at an affordable cost and made healthcare available to people on their doorstep.

The ECHP was started as a pilot PHC project of the Tulsi Chanrai Foundation with the objective being to develop a sustainable and replicable model PHC system in Eniosa. Another objective was to reduce morbidity and mortality amongst mothers and children and to prevent early childhood diseases. The ECHP area covers 11 larger villages with clinics and numerous surrounding hamlets. The project covers an area of approximately 25 km^2^ with an estimated population of 20 500 people. This article was derived from the larger assessment study and focuses on activities at nine of the clinics, excluding the base at Eniosa and the clinic at Atapa which was newly-established at the time of the study.

Indigenously-trained health workers, supervised by a doctor and a nurse-midwife based at Eniosa Base Clinic, set up and run the centres. The clinics are staffed by locally-selected ‘health workers’ who have finished their school certificate and have received a three-month pre-service training session and subsequent in-service training on a weekly basis. The main services provided by the clinics include quality pre- and post-natal care, clean and safe delivery, immunisation and growth monitoring of infants, nutrition interventions for malnourished children and general health promotion. The validation exercise was part of the 2002 evaluation exercise for the Foundation and the aim of this study was to test the accuracy of record keeping in PHC services in the nine clinics run by the ECHP in Ibadan, Nigeria.

## Research method and design

Eniosa community is located in the western part of Lagelu LGA, 15 km northeast of Ibadan, the Oyo State capital. The LGA is primarily rural and agricultural and has an estimated population of 69 000 based on the 1991 census. This assessment began with a review of clinic records. All the clinics started to offer full treatment in 1998 and the project was planned to expand to other States in Nigeria in 2001. The Eniosa project maintained records for each of the following: antenatal care (ANC) register, drug register, immunisation register, family visit diary, outpatient department (OPD) register, data summary sheets and an obstetric activities register. For example, from the ANC register, the total number of pregnant mothers who booked, attended ANC and delivered at the clinic was recorded on a tally sheet. From the drug register, the evaluation team made note of the actual number of drugs available, the number of drugs given out free-of-charge (mainly to small children under the age of one year) and the number sold to adults. The immunisation register provided the opportunity to identify how much of each antigen given out; this included the total number of children who started with the immunisation process and the actual number who were fully immunised. Being a retrospective descriptive analysis of the clinic records, a unique model in this assessment is the testing of the accuracy of the record keeping in the clinic. This was carried out by validating some of the activities carried out in each clinic. The validation exercise was performed by taking a sample of names from three of the registers, namely the ANC booking register, delivery register and immunisation register. The 10 most recent client names were extracted from each of the three registers. The clients’ villages were located with the assistance of the health worker in each clinic. Care was taken not to allow the health worker to influence the responses, so once the team got to the target village they excused the health worker and the researchers tried to locate the client (or parent of the client, in the case of immunisation) by asking for directions from co-villagers. Validation questions consisted of simply asking whether the client had utilised the particular service and asking for the time of utilisation. Accuracy of the dates reported by clients was also confirmed with physical evidence such as birth certificates or immunisation cards. All villages were visited at least once, but in those villages where the clients either traveled or were not available for the interview, two more attempts were made to visit them.

The validation exercise also consisted of trying to locate persons who should have been in the register during the period between the date of the tenth client's register entry and the current date. For example, if the 10 names on the birth register extended between November 2001 and April 2002, then the team would ask village leaders and members whether any other person had delivered a baby during that period. If a non-recorded event had happened, the person was located and information was obtained with regard to the place and date of booking, delivery or immunisation, as appropriate. On returning from the field, all questionnaires were checked and edited on a daily basis by the evaluation team. The data were coded and entered into the computer using the EPI INFO package version 6.04 for analysis. The data gathered were analysed and descriptive statistics was used in order to report the findings of the study.

## Results

### Results of review of records

**FIGURE 1:**
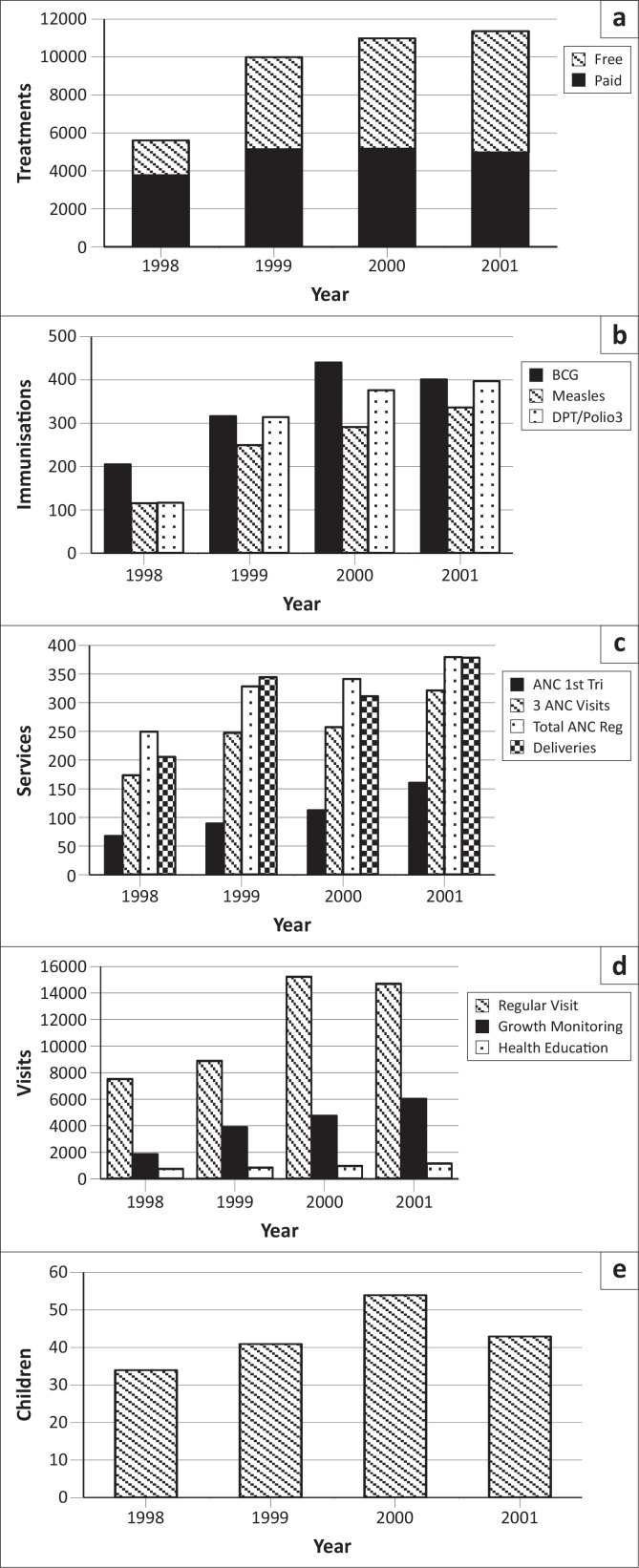
Review of records and utilisation thereof. (a) Treatments given; (b) Immunisations given; (c) Maternity services; (d) Family and village visits; (e) Malnourished children receiving food supplements.

Treatment was provided free for infants in the Eniosa system. Figure 1a reflects the treatment register and shows that whilst the number of patients paying for treatment was similar over the four-year period, the number of those receiving free treatment increased steadily. It should be noted that three of the clinics were not yet providing treatment in 1998. There were 5622 treatments recorded for 1998, of which 1865 (33%) were free. By 2001, this had increased to 11 376 treatments, of which 6420 (56%) were free. Figure 1b shows a steady increase in fully-immunised children (as found in the Eniosa registers) from 126 in 1998 to 337 in 2001. As can be seen, recording of BCG vaccinations increased through 2000, but dipped slightly in 2001. On the other hand, Measles vaccinations increased over the four-year period, from 116 to 337. The register reported DPT3 and Polio3 jointly, showing that they increased from 117 contacts in 1998 to 398 contacts in 2001. Details on DPT drop-out obtained from the coverage survey showed that whilst 86.1% children were administered with DPT1 and 71.9% children with DPT3, it appeared that 14.2% of children who started with their DPT immunisation did not complete third contact.

Antenatal Care and Safe Delivery are two of the major services provided by the ECHP. Figure 1c was drawn from the ANC and delivery registers in the Eniosa clinics. Ogunremi clinic was not yet functioning in 1998. The chart shows an increase in registration for all four indicators. Registration during the first trimester of pregnancy increased from 68 in 1998 to 161 in 2001. Likewise, the number of women who attended ANC at least three times rose from 174 in 1998 to 322 in 2001. Overall, 250 women booked for ANC services in 1998, whilst 380 did so in 2001. Finally, 206 women delivered in Eniosa facilities in 1998 and the number increased to 379 in 2001.

Eniosa clinics started offering Family Planning Services in 2000. The register shows that there were six condom packs distributed in 2000 and 101 in 2001. Twelve IUCD insertions were performed in 2000 and 19 in 2001. Six women received injectable contraception in 2000 versus 30 in 2001.

Family and/or village visits are a daily activity of the health worker. They check each house to find out if anyone has been sick, to update the birth register and to encourage the use of services. A register was also kept on family and/or village visits. Figure 1d shows that the number of families visited increased from 7544 in 1998 to 15 248 in 2000. The total number of visits that involved health education increased from 787 in 1998 to 1183 in 2001, with the overall percentage of visits devoted to health education being 6.6% in 1998 and 10.4% in 2000. Growth monitoring occurred during home visits and showed a steady increase from 1890 to 6050 over the period.

Eniosa Health Care has a supplemental nutrition programme for malnourished children under the age of five. These are identified through regular growth monitoring and village visits. Each mother receives four packets, the contents of which include dried cowpeas, cowpea flour for making *akara* (bean cakes), soybean flour for making soya milk and maize flour fortified with ingredients such as dried fish for making pap. This is expected to last for approximately one week and the mothers are encouraged to return to collect more until the child improves. A total of 172 children have benefited from this programme over the four-year period, as can be seen in Figure 1e.

Other registers were also reviewed. Table 1 highlights referrals made from 1998–2001. Two general types of referrals were made: those for complicated delivery and those for other medical or surgical reasons. Village-based clinics made their referrals to the Eniosa Base Clinic, whilst the Eniosa clinic made its referrals to appropriate specialist clinics and hospitals in Ibadan. Over the four-year period the number of referrals for delivery from village clinic to the base decreased from 23 to 12. Referrals for other reasons increased from 17 to 45 from 1998–2000 and then dropped to 29 in 2001. There were, on average, four referrals outside the Eniosa system over the four-year period.

**TABLE 1:** Patients referred from various village clinics to Eniosa community-health project base and from base to Ibadan hospitals (1998–2001).

Names of clinics	1998		1999		2000		2001	
			
	Delivery	Others	Delivery	Others	Delivery	Others	Delivery	Others
Village clinics	23	17	19	29	14	45	12	29
ECHP base	0	0	3	3	2	4	1	3

**Total**	**23**	**17**	**22**	**32**	**16**	**49**	**13**	**32**

ECHP, Eniosa Community-Health Project.

### Results from service utilisation

Table 2 shows the results of the register validation exercise. It was possible to trace 86% of those patients selected from the ANC register, 88.9% of those from the birth register and 81.1% of those from the immunisation register. All of these confirmed that they had in fact used the service specified. After asking in each village visited whether any additional people should have been registered within the dates selected for those found in the register, the interviewers found four people who should have been included for ANC, seven who had delivered, but were not in the register, and 13 who reported receiving immunisation but were not listed. Three of the unregistered ANC clients claimed to have registered, whilst one had not yet sought care. The birth register is filled out based on information obtained during home visits, since it includes all places of delivery, not only those occurring at Eniosa clinics. Of the 13 who were not included in the immunisation register, nine said that their child was vaccinated at an ECHP clinic, one went to a private clinic and three could not remember. Table 3 shows that most births occurred at home (45.3%), followed by Eniosa clinics (37.2%). Of the seven who had delivered but were not registered, only two (14.3% each) reported an assisted birth (traditional birth attendant [TBA] or ECHP staff).

**TABLE 2:** Register validation.

Registration status	ANC		Birth		Immunisation	
		
	*n*	%	*n*	%	*n*	%
Names selected	90	–	90	–	90	–
People traced	74	86.0	80	88.9	73	81.1
Traced and confirmed	74	100.0	80	100.0	73	100.0
Total found	78	–	87	–	86	–
Not registered	4	5.1	7	8.0	13	15.1

ANC, Antenatal care.

**TABLE 3:** Place of delivery.

Place delivered	In birth register		Not in register		Total	
		
	*n*	%	*n*	%	*N*	%
Home	34	43.0	5	71.4	39	45.3
ECHP	32	40.5	0	0	32	37.2
Church	8	10.1	1	14.3	9	10.5
Other clinics	5	6.3	0	0	5	5.8
On the road	0	0	1	14.3	1	1.2

**Total**	**79**	**91.9**	**7**	**8.1**	**86**	**100**

ECHP, Eniosa Community-Health Project.

This study documented the number of assisted deliveries in all places where the 86 registered- and unregistered births that are described in Table 3 occurred. Assisted delivery comprised those births where an ECHP health worker, a trained TBA or a health staff member of another clinic attended the birth. Overall, 58.1% of the births were attended by a trained attendant. All births in a clinic were delivered by a trained person, whilst only 28.0% of deliveries outside a clinic received the attention of a trained attendant. Both trained TBAs and ECHP staff visited homes for deliveries if necessary.

Multiple reasons were obtained from the women (41.9%) who delivered without trained assistance. Ten claimed they delivered unassisted because they went into labour after clinic hours or late at night. Six claimed that the health worker was not around. Five complained about distance or lack of transport. Three said it was because they delivered on a weekend or holiday, whilst two said that labour came on suddenly. Two more said they preferred to deliver at church. One each said that it was not her first experience, that she had not registered for ANC, that she did not tell anyone she was in labour and that she had delivered on the road on the way to the clinic. Five did not provide any explanation.

The registers also included a record of adverse events such as miscarriage, still births and deaths, as seen in Table 4. As with other register reviews, the year 1998 does not reflect those clinics that were yet to open fully. In absolute terms it is hoped that the 2001 figures reflect access to improved quality maternity services, as all child outcome measures for 2001 are lower than 1999. It was possible to make a calculation of infant mortality rate from the birth and death data available in the registers. Table 4 shows the proportion of recorded births that were delivered by a trained attendant and the infant mortality rate for the years 1998–2001. It should be recalled that the validation of the birth register identified that approximately 8% of births may not have been recorded. Due to their sensitive nature, child deaths were not validated, meaning that the infant mortality calculations in the table can only be called estimates.

**TABLE 4:** Birth records, Mortality estimates and Adverse events (1998–2001).

Activities	1998	1999	2000	2001	Total
**Review of Register of Adverse Events**					
Miscarriage	5 (12.5%)	14 (35.0%)	12 (30.0%)	9 (22.5%)	40
Stillbirth	2 (28.6%)	3 (42.8%)	1 (14.3%)	1 (14.3%)	7
Death at birth	3 (60.0%)	2 (40.0%)	0	0	5
Infant death	2 (5.7%)	11 (31.4%)	13 (37.1%)	9 (25.8%)	35
Maternal death	1 (50.0%)	0	0	1 (50.0%)	2
**Births and Infant Mortality Estimates**					
Births by trained attendant	135 (54.4%)	211 (64.5%)	186 (61.6%)	307 (81.0%)	839
Births by untrained attendant	113 (45.6%)	116 (35.5%)	116 (38.4%)	72 (19.0%)	417
Infant mortality estimates per 1000 live births	5 (20.2%)	13 (39.8%)	13 (43.0%)	9 (23.7%)	40
**Total births recorded**	**248**	**327**	**302**	**379**	**1256**

## Ethical considerations

On getting to the village, field staff sought the consents of the participants. As a normal practice in traditional society, the head of the village was met initially and the purpose of the visit was explained to him. The field staff proceeded to interview the respondents after the approval of the village heads. Field staff also explained to the respondents the purpose of the study, the duration of the interview and the benefit to be derived as a result of their participation. Participation was voluntary and respondents were made to know that they were free to withdraw from the interview at any time they wanted. The study involved little or no harm; however, the base clinic of ECHP had been prepared to take care of prospective participants whose participation, in any case, had had an adverse effect. All the questionnaires used to gather the data in this study were locked in a safe and kept in a place that is accessible only by the researchers.

## Trustworthiness

The instrument used for the study was pretested for standardisation and to ascertain its validity and reliability. The pretest took place amongst women of a similar or almost similar sociodemographic structure in a nearby community where ECHP was not in operation. At the end of the pretesting, responses gathered were considered in order to validate the content and to improve its standard. All these processes were put in place to ensure the trustworthiness of the study through validity and reliability.

## Discussion

This validation exercise of the ECHP employed a variety of methods to gather information and it should be stated that the detailed record keeping at both the facility- and central level was a most valuable resource for the current exercise. These records allowed a vision of programme activities and results over a four-year span. Previous assessments had also relied on these well-kept records. What was different about the ECHP assessment was the sampling of names and tracing them to their different villages for interviews regarding their use of the facility.

A review of the health-facility records showed a general increase in service utilisation between 1998 and 2001. Some of the increase between 1998 and 1999 was due to the opening of a new clinic, but overall the trend was still positive. The exception was a drop in BCG immunisations in 2001. As is evident from the immunisation programme, as reported in the Nigeria Demographic and Health Survey (NDHS) 1999,28 measles vaccinations occur less than other immunisations. Whilst this implies room for improvement in follow up, one should be encouraged by the survey findings that show a significantly lower drop-out rate amongst children in the ECHP area.

Home visits showed a slight drop in 2001. It is worth noting that throughout the years, few home visits were recorded as being for the purpose of health education. Clearly, many of the visits were ‘educational’ in general in that they encouraged utilisation of services, but these records show that the visits, which are generally well received by the villagers, could have a more specific educational agenda. A monthly plan for educational outreach could be developed for the programme.

A unique aspect of the assessment of ECHP was the register validation exercise. Basically, the records as they stand are accurate in that names listed as having utilised a service did in fact do so. This lack of ‘false positive’ service statistics is a testimony to the honesty of the health staff. What was discovered in the process of tracing the listed clients was that a few people who had obtained a service were not recorded. This ranged from 5.1% of ANC clients traced to 15.1% of children who had reportedly received immunisation. One can thus conclude that the names appearing in the register are likely to represent valid events, but that the registers did not actually capture all such events in the community.

Eight per cent of births that had occurred in the record-review period had not been recorded and, as expected, none had taken place at an ECHP facility. In general, the health workers during their home visits were able to keep track of non-facility-based births, but these had slipped through and affected the calculations of infant mortality, contributed to an underestimation of the births in the area and child-health needs and resulted in an over-estimation of maternity service utilisation, particularly the proportion who were delivered by a trained attendant. As it stands, two-fifths of births were delivered by an untrained attendant, which is slightly higher than NDHS (1999)^28^ reported for the southwestern zone of the country. The proportion delivered by a trained attendant showed a general increase over the period, whilst the estimated infant mortality using the register information shows a decrease.

It is suggested that even though immunisation completion rates and ANC utilisation are high, there is always room for improvement. Village health committees should be involved in tracing defaulters and in other outreach activities. Training of TBAs should continue with an effort at reducing the somewhat large proportion of births that are delivered by untrained attendants. Village visits should also be used as an opportunity to validate and update clinics records. It is ascertained that the high rate of positive validated data in the field was based on the high and total commitment of the Tulsi Chanrai Foundation staff; but yet further commitment is necessary in order to cultivate loyalty, improve performance and optimise cooperation amongst the community people. Finally, record-validation exercise should become an annual event and an in-service learning experience for the staff.

## Conclusion

This study concludes that the names appearing in the register are likely to represent valid events, but that the registers did not capture all such events in the community.
